# An experimental study investigating the effect of pain relief from oral analgesia on lumbar range of motion, velocity, acceleration and movement irregularity

**DOI:** 10.1186/1471-2474-15-304

**Published:** 2014-09-16

**Authors:** Jonathan M Williams, Inam Haq, Raymond Y Lee

**Affiliations:** Bournemouth University, School of Health and Social Care, Royal London House, Christchurch Road, Bournemouth, Dorset BH1 3LT UK; Brighton and Sussex Medical School, University of Brighton, Mayfield House, Falmer, BN1 9PH UK; Department of Life Sciences, Roehampton University, Whitelands College, Hoybourne avenue, London, SW15 4JD UK

**Keywords:** Pain relief, Low back pain, Lumbar, Kinematics, Inertial sensor, Biomechanics

## Abstract

**Background:**

Movement alterations are often reported in individuals with back pain. However the mechanisms behind these movement alterations are not well understood. A commonly cited mechanism is pain. The aim of this study was to investigate the effect of pain reduction, from oral analgesia, on lumbar kinematics in individuals with acute and chronic low back pain.

**Methods:**

A prospective, cross-sectional, experimental repeated-measures design was used. Twenty acute and 20 chronic individuals with low back pain were recruited from General Practitioner and self-referrals to therapy departments for low back pain. Participants complained of movement evoked low back pain. Inertial sensors were attached to the sacrum and lumbar spine and used to measure kinematics. Kinematic variables measured were range of motion, angular velocity and angular acceleration as well as a determining movement irregularity (a measure of deviation from smooth motion). Kinematics were investigated before and after administration of oral analgesia to instigate pain reduction.

**Results:**

Pain was significantly reduced following oral analgesia. There were no significant effects on the kinematic variables before and after pain reduction from oral analgesia. There was no interaction between the variables group (acute and chronic) and time (pre and post pain reduction).

**Conclusion:**

The results demonstrate that pain reduction did not alter lumbar range of motion, angular velocity, angular acceleration or movement irregularity questioning the role of pain in lumbar kinematics.

**Electronic supplementary material:**

The online version of this article (doi:10.1186/1471-2474-15-304) contains supplementary material, which is available to authorized users.

## Background

Low back pain (LBP) is a leading cause of disability and a major health and socioeconomic burden [1]. Persons with LBP commonly display alterations in their lumbar kinematics [2–9]. Alterations, in the form of deficits, have been determined in range of motion (ROM) [6–8], however the higher order kinematics, such as angular velocity and angular acceleration are also affected [2, 3, 5–11]. These alterations have been identified in studies comparing those with LBP to controls [4–11]. Furthermore prospective studies have also identified that individuals with relatively lower peak velocities and accelerations are at greater risk of LBP reporting [12, 13], as are those involved with occupational tasks requiring greater velocities [3]. Moreover in those individuals recovering from LBP the higher order kinematics have shown to be linked to the recovery or recurrence of pain [10, 13, 14]. Therefore lumbar kinematics may have a role in predicting LBP reporting but also lumbar kinematics may be altered by the presence of LBP. Despite these frequent observations, the underlying mechanisms causing these alterations are not well understood.

Previous authors have suggested these alterations are driven by pain [15, 16]. In order to investigate this theory, experimentally induced pain models have been adopted. Experimental studies investigating the effects of induced pain on previously pain free individuals show that pain results in a reduction of trunk ROM and velocity during forward bending and a reduction in trunk acceleration during walking [17, 18]. However authors have demonstrated that experimentally induced pain fails to alter trunk-pelvis rotation coupling or phase of gait during level walking [19, 20]. Whilst these studies provide some insight into the interaction between pain and kinematics they do not investigate other spinal movements, such as backward bending, side bending or twisting. Moreover induced pain models commonly produce pain which is constant in nature with little deviation except for a gradual reduction over time. This pain behaviour fails to mimic the movement evoked pain often presenting in a clinical environment [21].

In order to study the relationship between pain and kinematics previous authors have also utilised an induced pain reduction model in a sample of individuals with LBP. This has demonstrated that pain reduction results in significant gains in ROM [22, 23], however the gains were not universal across all movements and the magnitude of gain was small. Using similar methods, changes in movement velocity have been demonstrated during sit-to-stand and spinal movements [24, 25]. This alternative model investigates the role of pain in movement alterations by determining the effects of experimentally relieving pain associated with altered movement. Therefore it may be clinically useful to investigate the effects of pain reduction to determine the relationship between pain and kinematics. However in these previous studies it cannot be determined if the changes are attributable to pain reduction in isolation as the methods used failed to target pain in isolation, instead using either ill-defined manual therapy [25] or superficial heat [24], which are known to affect the properties of musculoskeletal tissues [26, 27]. Therefore the effect of manipulating pain in isolation remains unclear in patients with LBP.

The purpose of this study was to investigate the immediate effects of pain reduction, from oral analgesia, on lumbar ROM, angular velocity, angular acceleration and movement irregularity in patients with ALBP and CLBP.

## Methods

A prospective, cross-sectional, experimental repeated-measures design was used. Forty volunteers with LBP were recruited from local physiotherapy and chiropractic clinics over a six month period. Participants were screened by a physiotherapist for inclusion and exclusion criteria (Table 1) and were divided into one of two groups according to duration of pain (Table 2). The severity of the pain was rated using a visual analogue scale (0 to 100 mm) where participants were asked to mark their average pain over the preceding week, and fear of movement was evaluated using the Tampa Scale of Kinesiophobia questionnaire (TSK). All volunteers with LBP were required to report pain during at least three of the tested movements and therefore were classified as having movement evoked or ‘mechanical’ LBP.

**Table 1:** **Inclusion and exclusion criteria**

Inclusion	Exclusion
Pain confined to between lower ribs and inferior gluteal folds	History of tumors
	Spinal fractures
Movement evoked pain	
Aged 18–55 years old	
Seeking healthcare for LBP	
Acute – Pain present for less than 3 weeks on a history of no pain for at least 12 months	Surgery
	Neurological signs or symptoms
Chronic – Pain present on at least 3 days per week for at least 52 weeks	Rhuematological or Neurological disease
	Known spinal deformity

**Table 2:** **Participant demographics (Mean (sd))**

	ALBP	CLBP	t-test
Male/Female	11/9	11/9	
Age (years)	42.7 (6.8)	36.6 (10.8)	0.08
Height (cm)	172.9 (11.3)	173.6 (11.2)	0.70
Weight (kg)	82.6 (16.6)	83.7 (16.1)	0.55
BMI (kg/m^2^)	27.5 (4.0)	26.2 (4.1)	0.43
TSK	39.0 (4.8)	38.9 (6.9)	0.85
VAS (100 mm scale)	62.2 (16.6)	46 (22)	0.02
Duration	12.3 (6.7) days	9.4 (7.4) years	<.001

ALBP; acute low back pain, CLBP; chronic low back pain, BMI; body mass index, TSK; tampa scale of kinesiophobia, VAS; visual analogue scale.

A sample size calculation was completed with power set at 0.8, alpha at 0.05 and a modest effect size 0.7. The effect size was calculated from aiming to achieve a 3° change in ROM with a standard deviation of 4.5° based on previous work [28].

All participants were supplied with an information sheet, following which they gave informed consent and their rights were protected. The study was approved by the National Research Ethics Service of the National Health Service (08/H1111/38).

Two wired 3DM-GX3-25 inertial sensors were used to measure lumbar kinematics (GX3-25, Microstrain, VT, USA). Each sensor contained three integrated sensing elements including gyroscopes (±300°/s), accelerometers (±5 g) and magnetometers and provides absolute orientation to a company reported accuracy of 0.5 degrees [29]. One sensor was fixed over the S1 spinous process and the second over the L1 spinous process. The sensors were attached using double sided tape with the wires secured to the trunk so as to prevent them erroneously moving the sensor. The sensors were connected to a purpose built datalogger and software (THETAmetrix, Waterlooville, UK) with data captured at 100Hz. Lumbar spine movements were calculated from the relative orientations between the L1 and S1 sensor derived from direction cosine matrices using mathematical methods described in detail elsewhere [30, 31]. Flexion, left side bending and left rotation were considered positive and the opposite movements negative.

Participants were requested to abstain from any medication on the day prior to and of testing. Three trials of forward and backward bending, side-bending, twisting and lifting were completed. No constraints were placed on any of the movements. This ensures the movements were completed naturally, better reflects the clinical situation and mirrors functional tasks. The object to be lifted was a box with dimensions 460 × 260 × 300 mm which weighed 3 kg. The magnitude of the worst pain evoked by the movements was measured using a visual analogue scale completed following each of the three movement trials.

Participants were then requested to self-administer their chosen oral analgesia and given a break of between 45–60 minutes after which the movements were repeated. No restrictions were placed on the type of analgesia used as the aim of this study was to investigate the effects of pain reduction not to determine the efficacy of a particular medication. A medication approach was used to manipulate pain, as it is believed this would isolate the effects to pain and the use of self-administered usual medication was the most convenient and ethically acceptable. The sensors remained attached throughout the experiment. In order to explore the effects of pain reduction on movement evoked pain, only those movements which evoked pain on initial testing were analysed thus removing the confounding factors of pain free movements.

**Figure 1:**
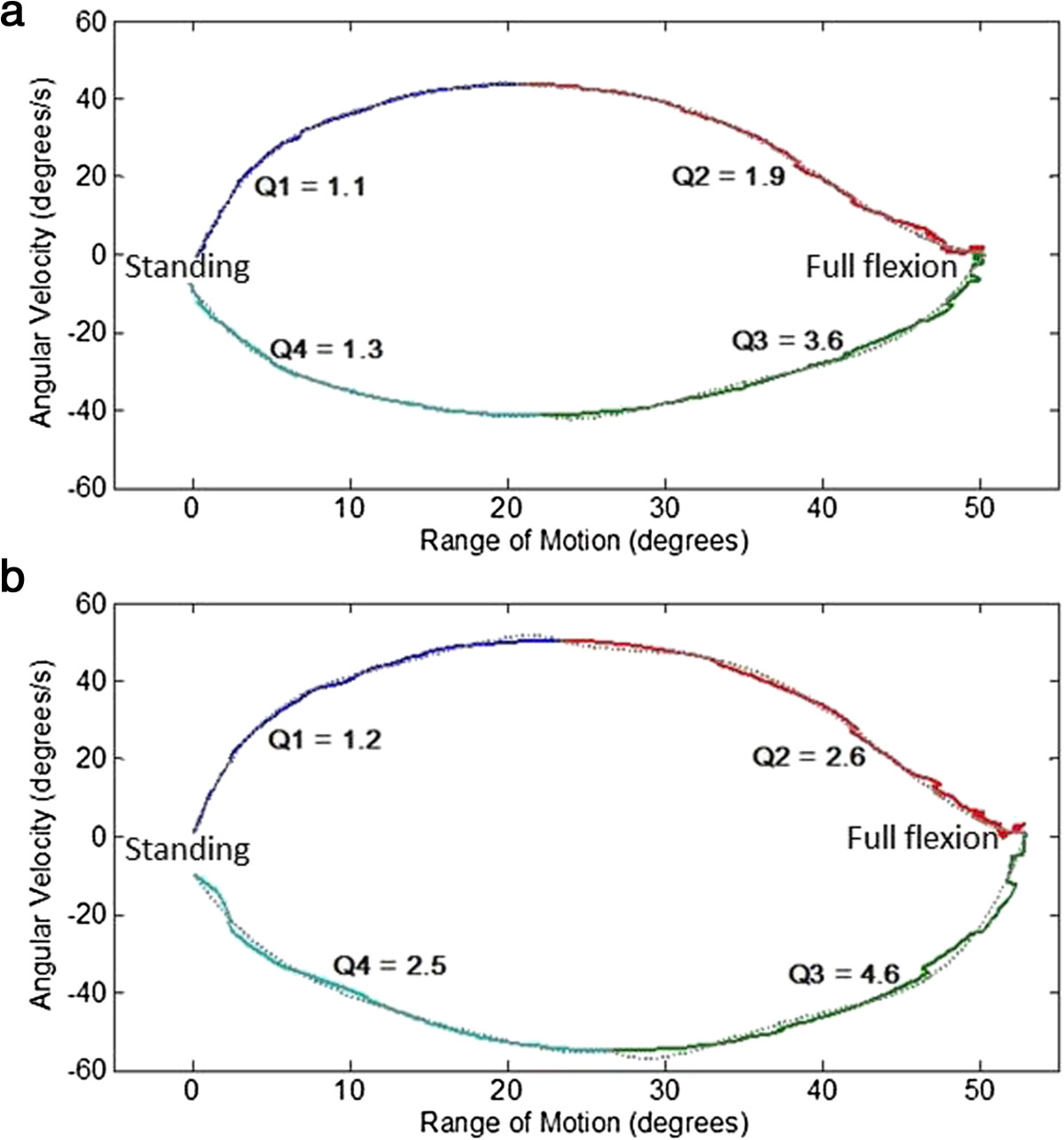
**Spatial plots annotated with movement irregularity scores of a single participant for the movement of flexion.** No effect of pain relief on movement irregularity is demonstrated. **(a)** Flexion trial VAS = 24; **(b)** Flexion trial VAS = 0.

All processing was completed using Matlab (Mathworks 2008b, Natick, MA, USA). The movement-time data for each movement were determined and differentiated to yield velocity and acceleration using a method described previously [32]. The peak range of motion (ROM), peak angular velocities and peak angular accelerations for the primary planar movements only were calculated, which for lifting was considered to be in the sagittal plane. Such processing methods have demonstrated good-to-excellent repeated measures reliability with small mean absolute errors between measurements [32]. To aid interpretation of the change in variables following pain reduction, differences in kinematics were sign normalised so increases in the variable (i.e. greater ROM or greater velocity/acceleration) were assigned positive values and decreases in the variable were assigned negative values. Movement irregularity was also determined by plotting and quantifying the spatial relationships between angular velocity and movement curves. This was achieved using a method described previously but is briefly described below [32]. The angular velocity-movement plot was sectioned into four quartiles based on the points of zero ROM and peak velocity. Each quartile was fitted with a fourth order polynomial and root mean square error between the actual and fitted data was used as a measure of movement trajectory irregularity (Figure 1a). Such processing methods have demonstrated moderate-to-good repeated measures reliability and small mean absolute differences between repeated movements [32].

The frequency of responders to pain reduction for each movement in each group was determined, where responders were defined as those individuals who increased the kinematic variables by more than a pre-set threshold. The thresholds for flexion and lifting were set at 3°, 4°s^−1^, 8°s^−2^ for ROM, velocity and acceleration respectively. The thresholds for extension and side bending were 2°, 3°s^−1^, 6°s^−2^ and for rotation were 1°, 2°s^−1^, 4°s^−2^ for ROM, velocity and acceleration respectively. These thresholds were selected with reference to the natural variation of repeated movements demonstrated in an earlier study [32].

Statistical analysis was completed using SPSS 20. t-tests were used to compare demographics. A multivariate ANOVA (MANOVA) was completed to determine the effects of group and time on the kinematics, using two independent variables, group (ALBP and CLBP) and time (pre and post pain reduction) and five dependent variables (ROM, positive angular velocity and angular acceleration and negative angular velocity and angular acceleration). A MANOVA model was chosen over conducting numerous ANOVAs to reduce type I error, given the various dependent variables were conceptually related to each other. Movement irregularity pre and post pain reduction from oral analgesia was compared for each quartile using paired t-tests or Wilcoxon signed rank test when normality could not be assumed. A comparison between groups for frequency of responders was completed using the Chi-squared test. Furthermore a correlation between the degree of pain reduction and magnitude of kinematic change was determined for each variable and group. For all statistical tests significance was set at 0.05. We confirm that our research has, where appropriate, adhered to the STROBE guidelines.

## Results

There were no significant differences between the groups at baseline except for duration and severity of pain. There were significant reductions in the degree of movement evoked pain following oral analgesia for both groups and all movements demonstrating that oral analgesia provided significant reductions in pain (Table 3). Analgesia choices were similar amongst the groups with the exception of the ALBP group favouring a combination of analgesia and anti-inflammatory medication. Common choices were Ibuprofen, Paracetamol, Co-codamol, Naproxen and Dicloflex. There were no significant differences between the groups for the amount of evoked pain experienced, with the exception of the lifting where the CLBP group reported greater pain (20.6 mm) (Table 3).

**Table 3:** **Degree of evoked pain before and after analgesia (Mean (sd) VAS measured on a 100 mm scale)**

	ALBP Pre	ALBP Post	t-test	CLBP Pre	CLBP Post	t-test
Flexion	41.1 (24.4)	20.7 (26.4)	<0.01	39.6 (20.4)	3.0 (9.5)	<.001
Extension	41.2 (19.6)	17.6 (19.8)	<.001	40.9 (24.2)	17.2 (25.0)	<.001
Side-Bending	40.4 (23.9)	18.3 (22.8)	<.001	35.2 (21.9)	10.0 (22.3)	<.001
Rotation	38.5 (21.2)	9.7 (17.7)	<.001	37.0 (21.9)	7.8 (22.3)	<.001
Lifting	31.6 (17.8)	15.2 (22.0)	<0.01	52.2 (25.0)	28.3 (27.0)	<.001

ALBP; acute low back pain, CLBP; chronic low back pain, VAS; visual analogue scale.

**Figure 2:**
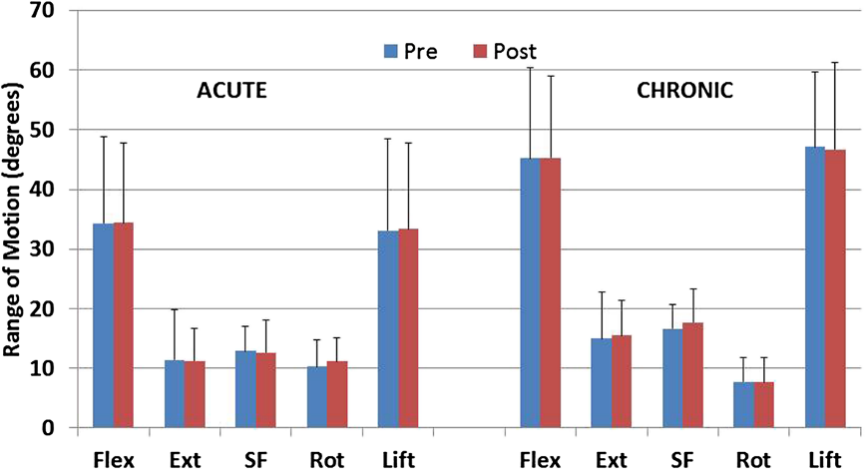
**Lumbar range of motion for each group before and after pain relief.** Notes; Pre, before pain relief; Post, after pain relief; ALBP, acute low back pain; CLBP, chronic low back pain; Flex, flexion; Ext, extension; SF, side flexion; Rot, rotation.

**Figure 3:**
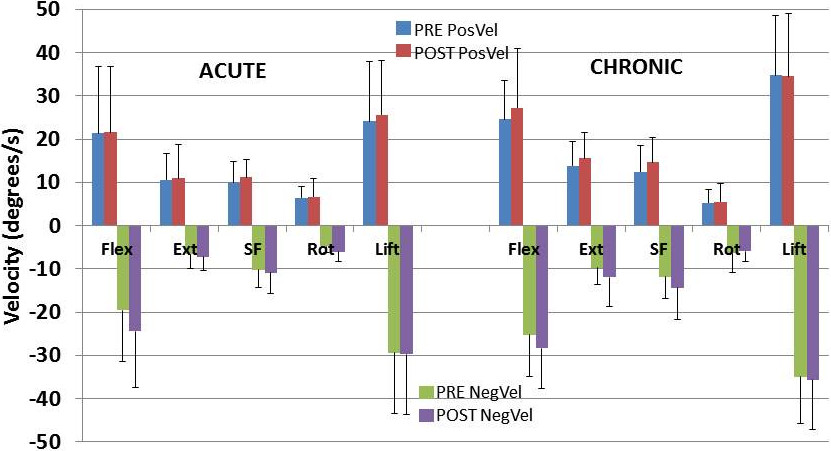
**Lumbar velocity for each group before and after pain relief.** Notes; Pre, before pain relief; Post, after pain relief; ALBP, acute low back pain; CLBP, chronic low back pain; Flex, flexion; Ext, extension; SF, side flexion; Rot, rotation; PosVel, positive velocity, NegVel, negative velocity.

**Figure 4:**
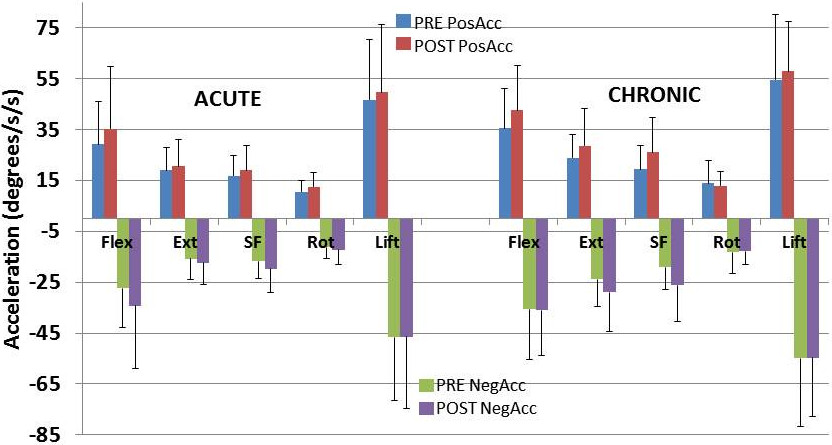
**Lumbar acceleration for each group before and after pain relief.** Notes; Pre, before pain relief; Post, after pain relief; ALBP, acute low back pain; CLBP, chronic low back pain; Flex, flexion; Ext, extension; SF, side flexion; Rot, rotation; PosAcc, positive acceleration; NegAcc, negative acceleration.

The group kinematics prior to and following pain reduction are presented in Figures 2, 3 and 4 for ROM, velocity and acceleration respectively. Kinematic changes following pain reduction are presented in Table 4.

**Table 4:** **Change in kinematic variables (mean (sd)) in response to pain relief**

Flexion	ALBP	95% CI	CLBP	95% CI
Mean change in ROM (^o^)	0.1 (4.3)	−2.3 – 2.6	0.1 (6.6)	−4.9 – 4.6
Mean change in Positive Velocity (^o^s^−1^)	0.3 (5.2)	−3.2 – 2.6	2.6 (4.4)	−0.6 – 5.7
Mean change Negative Velocity (^o^s^−1^)	4.8 (4.7)	2.2 – 7.3	1.1 (6.1)	−5.5 – 3.2
Mean change Positive Acceleration (^o^s^−2^)	6.1 (12.3)	−12.9 – 0.7	7.1 (8.1)	1.3 – 12.8
Mean change Negative Acceleration (^o^s^−2^)	6.9 (11.7)	0.4 – 13.4	0.5 (7.9)	−6.2 – 5.1
Extension				
Mean change in ROM (^o^)	−0.3 (2.2)	−0.9 – 1.5	1.0 (2.8)	−0.6 – 2.6
Mean change in Positive Velocity (^o^s^−1^)	0.3 (4.4)	−2.8 – 2.0	1.8 (4.0)	−0.5 – 4.1
Mean change Negative Velocity (^o^s^−1^)	0.6 (1.9)	−0.5 – 1.6	2.1 (4.8)	−4.9 – 0.6
Mean change Positive Acceleration (^o^s^−2^)	1.7 (9.6)	−6.8 – 3.4	4.8 (8.4)	−0.1 – 9.7
Mean change Negative Acceleration (^o^s^−2^)	1.8 (9.2)	−3.2 – 6.7	5.1 (8.0)	−9.8 – -0.5
Side-bending				
Mean change in ROM (^o^)	−0.3 (1.6)	−0.3 – 0.9	1.0 (2.0)	0.3 – 1.9
Mean change in Positive Velocity (^o^s^−1^)	1.1 (3.2)	−2.4 – 0.1	2.1 (3.0)	0.9 – 3.4
Mean change Negative Velocity (^o^s^−1^)	0.7 (2.9)	−0.4 – 1.9	2.7 (3.3)	1.3 – 4.0
Mean change Positive Acceleration (^o^s^−2^)	2.1 (7.5)	−5.1 – 0.8	6.7 (7.1)	3.8 – 9.7
Mean change Negative Acceleration (^o^s^−2^)	3.1 (8.6)	−0.3 – 6.5	6.9 (9.6)	3.0 – 10.9
Rotation				
Mean change in ROM (^o^)	0.8 (2.0)	−2.1 – 0.5	−0.2 (2.9)	−1.7 – 1.3
Mean change in Positive Velocity (^o^s^−1^)	0.2 (2.0)	−1.5 – 1.1	0.0 (2.1)	−1.1 – 1.1
Mean change Negative Velocity (^o^s^−1^)	1.2 (1.7)	0.1 – 2.3	−0.7 (3.1)	−2.3 – 0.9
Mean change Positive Acceleration (^o^s^−2^)	1.8 (4.8)	−4.9 – 1.2	−1.4 (7.8)	−5.5 – 2.6
Mean change Negative Acceleration (^o^s^−2^)	1.1 (4.4)	−1.7 – 3.9	−0.7 (6.8)	−4.2 – 2.8
Lifting				
Mean change in ROM (^o^)	0.2 (5.5)	−3.1 – 3.5	0.4 (4.5)	−2.5 – 3.3
Mean change in Positive Velocity (^o^s^−1^)	1.5 (5.7)	−4.9 – 1.9	−0.2 (9.7)	−6.3 – 5.9
Mean change Negative Velocity (^o^s^−1^)	0.3 (5.2)	−2.9 – 3.4	−0.6 (4.8)	−3.7 – 2.4
Mean change Positive Acceleration (^o^s^−2^)	3.0 (10.9)	−9.6 – 3.6	3.2 (16.3)	−7.1 – 13.6
Mean change Negative Acceleration (^o^s^−2^)	0.0 (13.0)	−7.9 – 7.8	3.3 (12.6)	−4.7 – 11.4

ROM, range of motion; ALBP, acute low back pain; CLBP, chronic low back pain.

Note: A positive value represents an increase in the specific variable, negative a reduction.

There were no significant effects on the kinematic variables before and after pain reduction (F = 1.23, p = 0.29; F = 1.68, p = 0.14). Furthermore there was no interaction between the variables group and time (pre/post pain reduction) (F = 0.11, p = 0.99). These results therefore suggest that neither reduction of pain nor chronicity of pain had any effect on lumbar kinematics. As no significant effect was determined post hoc testing was unnecessary.

Pain reduction had no effect on movement irregularity for each quartile of any movement tested (Figure 1), with the exception of quartile 3 for flexion in the ALBP group (reduction in movement irregularity (mean difference 1.1, p = 0.02)), and quartile 3 for side bending in the CLBP group (increase in movement irregularity (mean difference 1.2, p = 0.05 side bending). These changes in irregularity scores are small and within the realms of natural variation [32].

Chi-squared testing revealed no significant difference in the number of responders between the groups for ROM (χ^2^ = 2.83, p = 0.09), angular velocity (χ^2^ = 0.01-1.88, p = 0.17-0.91), angular acceleration (χ^2^ = 0.00-0.69, p = 0.41-0.97) or movement irregularity (χ^2^ = 0.03-3.60, p = 0.06-0.88).

Overall no significant correlation could be established for magnitude of pain reduction and magnitude of kinematic change for the chronic LBP group (all absolute r-values < 0.58; p-values > 0.05). However the acute LBP group demonstrated a significant correlation for pain reduction and negative velocity change for flexion (r = 0.53; p = 0.04) and left side bending (LSF) (r = 0.54; p = 0.05) as well as positive acceleration for LSF (r = −0.77; p > 0.001). All other correlations in the acute LBP did not reach significance. These weak positive correlations demonstrate a weak relationship between decreasing pain and increasing velocity for flexion, ROM for LSF and acceleration for LSF.

## Discussion

The present study investigated the effect of pain reduction, from oral analgesia, on lumbar kinematics in patients with acute and chronic LBP. The experimental pain-relief model was unable to provide complete relief of pain. The observed degree of pain reduction was not only statistically significant (p < 0.003) but also clinically significant as it exceeds the recommended minimally important change in VAS of 15 mm or 30% change in baseline value [33]. Such significant change in pain enables the role of pain in altered movements of the spine to be investigated.

The findings of the present study demonstrate that pain reduction did not affect lumbar kinematics in individuals with acute or chronic back pain. Therefore there was no overall systemic effect across the groups as a whole. The findings are consistent with some of those within the literature and in contrast to others using similar experimental pain reduction models. In a similar sample of individuals with acute and chronic LBP, analgesia induced pain-reduction failed to increase spinal curvature during flexion, extension and lifting [5]. In a CLBP sample, Lilius et al. [22] demonstrated no gains in flexion, extension and rotation following a facet joint injection. Moreover, Davis and Kotowski [25] also in patients with CLBP, reported no significant gains in all ranges of motion following pain reduction, which are in agreement with the current study. However our findings and those of the above studies are in contrast to some other studies [25, 24]. Davis and Kotowski [25] reported a significant increase in lateral bending angular velocity and angular acceleration as well as rotation angular velocity and angular acceleration during cardinal plane spinal movements. Furthermore Simmonds [24] demonstrated a significant increase in sit-to-stand velocity following pain reduction. These contrasts may be explained by the movement protocol, which utilised movement completed as fast as possible [25] rather than self-selected, as in this study. Indeed during recovery from LBP, links have been established between improvements in movements performed as fast as possible and the reduction of pain [10, 14]. Therefore it may be possible that pain reduction affects the individual’s ability to move the spine at greater velocity as opposed to resulting in a shift of self-selected velocities, however this is something which requires further investigation.

Differences may also be due to the experimental pain reduction method used. In the case of Simmonds [24] superficial heating was used which may influence factors other than pain, such as the compliance of musculoskeletal tissues [26, 27]. These differences highlight the unique methodology used in the current study – simple oral medication induced pain reduction, which avoids soft tissue changes likely to be associated with other methods. Although it should be noted that the medications used are likely to have a systemic bio-physiological and chemical effect, the significance of which is not known. Moreover it is acknowledged that, as some of the individuals were likely routinely taking such medications, such a method may not offer a novel pain reduction stimulus to the individuals with LBP. However, this method is believed to provide a more specific manipulation of pain, rather than altering the mechanical properties of tissues. Improvements noted in other studies may be the result of biomechanical or neural changes in response to the chosen non-specific experimental pain reduction method.

The results demonstrate that pain reduction had minimal effect on the degree of movement irregularity as displayed though the use of spatial plots. Whilst it is open for debate whether greater movement irregularity should be interpreted as an impairment of spinal function, this study did not demonstrate changes in either direction (greater or lesser irregularity) in response to pain reduction. The use of spatial plot analysis has been little studied in relation to patients with LBP and these results are the first to investigate the effect of pain reduction on movement irregularity.

The current study suggests there may be variability in the role of pain as the response to pain reduction was not uniform across all individuals; however group comparisons of the frequency of these responders did not show a significant difference between the ALBP and CLBP group. These results suggest that individuals with ALBP or CLBP are just as likely to respond to pain reduction and that neither group is likely to be more ‘sensitive’ to pain reduction. There was a trend towards the ALBP group demonstrating a greater ratio of responders to non-responders for the measurement of movement irregularity especially within quartiles 2 and 3 of the movement trajectory compared to the CLBP group, however this did not reach significance. It could also be argued that the variability in response may be due to the types and dosages of medications used in the current study.

The lack of effect observed in the current study may be due to fear of movement commonplace in individuals with LBP [33, 34]. Lumbar movement has been shown to be affected by pain related fear and this may explain the lack of change observed in the current study [35–37]. High fear has demonstrated reduced lumbar excursion during reaching tasks at both preferred and fast speeds [35, 37]. This is in contrast to Thomas et al. [36] who using TSK to measure fear of movement demonstrated no such relationship was evident for lumbar excursion during self-selected speed reaching tasks. Participants in the current study did not demonstrate particularly high fear of movement and no correlations were established using TSK scores. Therefore it remains unclear as to the direct relationship between fear and lumbar kinematics, however it remains plausible that this may explain the lack of effect seen in the present results.

The lack of effect on kinematics could also be due to the small amount of pain reduction experienced from oral analgesia. It is not known what effect total pain relief would have on lumbar kinematics. Furthermore only the immediate effect of pain reduction was investigated. Therefore the results may reflect the need for the ‘system’ to experience less or no pain for longer in order to cause an alteration in trunk function. It is not clear if the same results would be replicated if the pain reduction was continued over a period of time. This learning effect was observed by Moseley and Hodges [38] in relation to induced pain and muscle function where repeated painful stimulus over seventy repetitions slowly resulted in changes in trunk muscle function and its gradual reversal following removal of the pain stimulus. The current study utilised only three movement trials and there was no indication of pattern between the first to the third trial, however it is possible that pain reduction over a longer time frame may yield different results.

It could be argued that this sample demonstrated movements which were unimpaired and matched their pre-pain movement patterns. In this scenario no change following the pain reduction would be expected. However the kinematics displayed by each group were significantly less than individuals without back pain [2, 39–42]. This could have been further explored by the addition of a control group, however this was deemed unnecessary as the aim of the study was not to test the efficacy of an intervention but rather investigate the effects of an experimental pain reduction model on lumbar kinematics.

The results of this study are limited to the immediate effects of pain relief, in an attempt to remove potential confounding variables that might influence the results. This meant the effects of analgesia on kinematics over time were not investigated. The sample size was limited and constrained to those with LBP confined to the back, therefore extrapolation to other types of LBP, such as radiculopathy, is not possible. The sample was formed of individuals with acute and chronic non-specific LBP. The individuals were defined as having mechanical or movement evoked LBP, however it is acknowledged that this may still represent a heterogeneous sample of individuals with back pain. It is not known whether a stricter classification of non-specific back pain would yield similar results. The current study was limited to spinal movements and the findings might not be applicable to other tasks.

## Conclusions

The administration of simple oral analgesia resulted in significant pain reduction but this did not directly lead to changes in lumbar kinematics in individuals with ALBP or CLBP. The relief of pain also had little effect on movement irregularity. Variability in response was observed, and the frequency of positive response was no different for patients with acute or chronic LBP. There was no correlation between the magnitude of pain reduction and degree of change in lumbar kinematics.

## Authors’ original submitted files for images

Below are the links to the authors’ original submitted files for images. Authors’ original file for figure 1 Authors’ original file for figure 2 Authors’ original file for figure 3 Authors’ original file for figure 4

## Abbreviations

LBP: Low back pain
ALBP: Acute low back pain
CLBP: Chronic low back pain
ROM: Range of motion
TSK: Tampa scale of kinesiophobia
mm: Millimetres
kg: Kilograms
MANOVA: Multivariate analysis of variance
LSF: Left side flexion.
