# Supplementary figures and images for: An experimental study investigating the effect of pain relief from oral analgesia on lumbar range of motion, velocity, acceleration and movement irregularity
Source: BMC Musculoskelet Disord. 2014 Sep 16;15:304. doi: 10.1186/1471-2474-15-304 (PMC4171543; doi:10.1186/1471-2474-15-304)

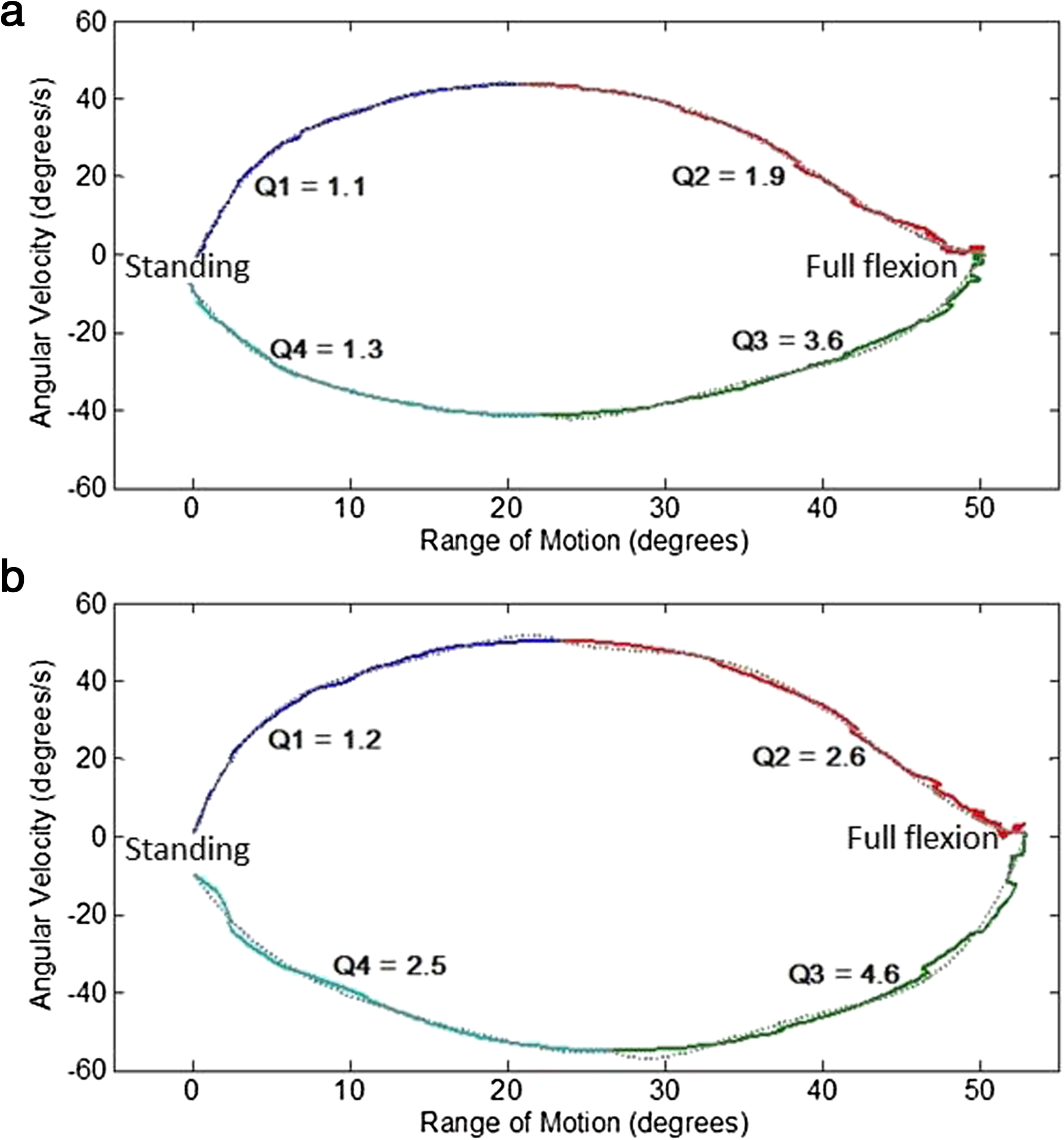

Supplement: Supplementary file 1 — Authors’ original file for figure 1 [file 12891_2014_2244_MOESM1_ESM.tiff]

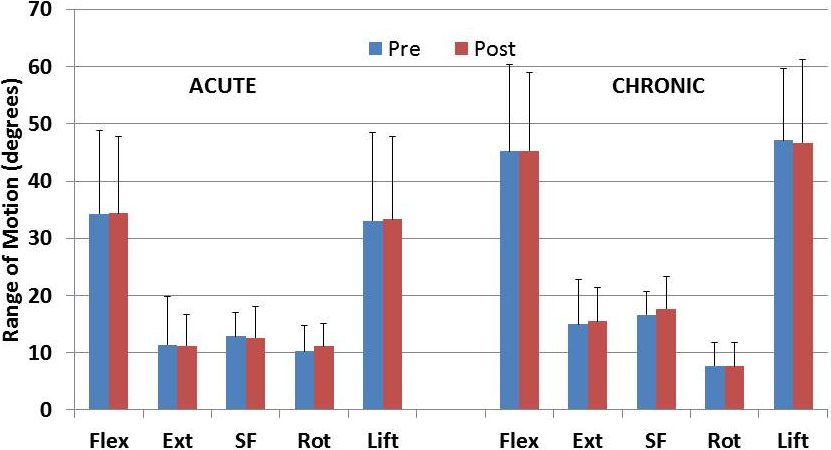

Supplement: Supplementary file 2 — Authors’ original file for figure 2 [file 12891_2014_2244_MOESM2_ESM.tif]

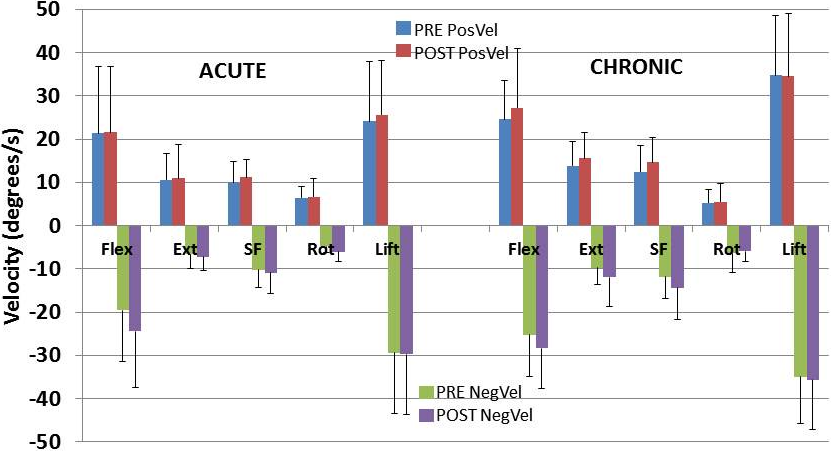

Supplement: Supplementary file 3 — Authors’ original file for figure 3 [file 12891_2014_2244_MOESM3_ESM.tif]

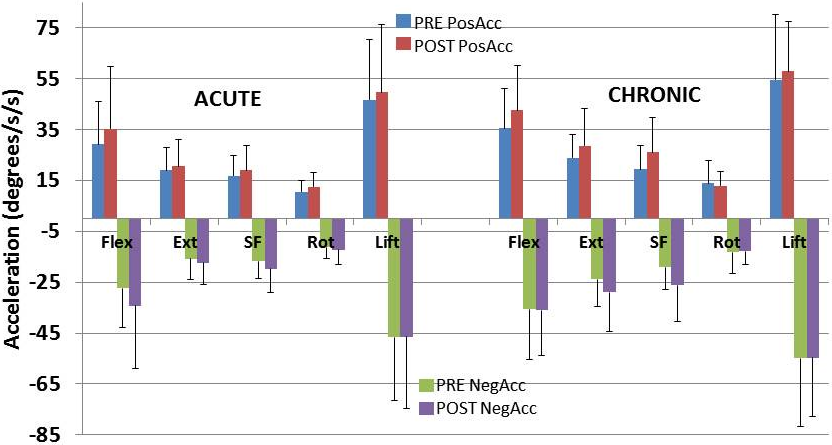

Supplement: Supplementary file 4 — Authors’ original file for figure 4 [file 12891_2014_2244_MOESM4_ESM.tif]
